# Supplementary material for: Content-rich biological network constructed by mining PubMed abstracts
Source: BMC Bioinformatics. 2004 Oct 8;5:147. doi: 10.1186/1471-2105-5-147 (PMC528731; doi:10.1186/1471-2105-5-147)
Supplement: Additional File 2 — The original results of the above study (non-essential files are deleted to keep the file size under the limit set by BMC bioinformatics). [file 1471-2105-5-147-S2.bz2 › chilibotAdditionalFile2/dip10/197ID8652557E680/html/ERCC3_GTF2H1.html]

 


 **ERCC3** and **GTF2H1** 
  
Found 427 abstracts in PubMed, retrieved 10.  
 

 What does Google say? 
 PDF only 
| .edu only 

---

**Interactive relationship** (e.g. stimulation, inhibition, etc)

**Neutral relationship**- Analyses of AR mediated transcription suggest that the orchestrated interaction of AR with the transcription factors IIF TFIIF and IIH TFIIH  [ **GTF2H1** ]   [ **ERCC3** ] , and positive transcription elongation factor b P TEFb, may increase efficiency of transcriptional elongation from the androgen target genes, such as prostate specific antigen PSA.  Ref: 12648523 J Steroid Biochem Mol Biol, 2003
- Recent advances have shed some light on the structure of these factors, how conformational changes and intramolecular interactions regulate activity, and have revealed an expanded role for TFIIH  [ **GTF2H1** ]   [ **ERCC3** ]  in nuclear transcription.  Ref: 12672487 Curr Opin Genet Dev, 2003
- Moreover, when XPD mutations prevent interaction with the p44 subunit of TFIIH  [ **GTF2H1** ]   [ **ERCC3** ] , transactivation directed by certain nuclear receptors is inhibited, regardless of TTD versus XP phenotype, thus explaining the overlapping symptoms.  Ref: 12820975 Mol Cell, 2003
- General transcription factor IIH TFIIH  [ **GTF2H1** ]   [ **ERCC3** ]  consists of nine subunits cyclin dependent kinase 7 Cdk7, cyclin H and MAT1 forming the Cdk activating kinase or CAK complex, the two helicases Xpb  [ **ERCC3** ]  Hay and Xpd, and p34, p44, p52 and p62 refs 1 3.  Ref: 12853965 Nature, 2003

**Non-interactive relationship** (e.g. studied together, co-existance, homology, etc.)

- As the kinase subunit of TFIIH  [ **GTF2H1** ]   [ **ERCC3** ] , Cdk7 participates in basal transcription by phosphorylating the carboxy terminal domain of the largest subunit of RNA polymerase II.  Ref: 12853965 Nature, 2003
- Together with recent reports linking TFIIH  [ **GTF2H1** ]   [ **ERCC3** ]  in regulating NR function, our findings provide new data to further support an important link between DNA repair proteins and nuclear receptor function.  Ref: 12874288 J Biol ChemJ Biol Chem, 2003
- We also show that TFIIH  [ **GTF2H1** ]   [ **ERCC3** ]  from TTD patients, but not from XP patients, exhibits a significant in vitro basal transcription defect in addition to a reduced intracellular concentration.  Ref: 12820975 Mol Cell, 2003
- Set1 association depends upon the TFIIH  [ **GTF2H1** ]   [ **ERCC3** ]  associated kinase that phosphorylates the Pol II C terminal domain CTD and mediates the transition between initiation and elongation, and Set1 interacts with the form of Pol II whose CTD is phosphorylated at serine 5 but not serine 2.  Ref: 12667453 Mol Cell, 2003
- The results also showed that dispersal of recruited XPC and TFIIH  [ **GTF2H1** ]   [ **ERCC3** ]  from DNA damage SNS occurs within a short period after DNA damage.  Ref: 12713809 DNA Repair Amst , 2003
- Mutations in these residues also affected the ability of hTFIIE beta to stimulate TFIIH  [ **GTF2H1** ]   [ **ERCC3** ]  mediated phosphorylation of the carboxy terminal heptapeptide repeats of the largest subunit of Pol II.  Ref: 12665589 Mol Cell Biol, 2003
- Here we show that the Drosophila TFIIH  [ **GTF2H1** ]   [ **ERCC3** ]  component Xpd negatively regulates the cell cycle function of Cdk7, the CAK activity.  Ref: 12853965 Nature, 2003
- By contrast, recruitment of both XPC and TFIIH  [ **GTF2H1** ]   [ **ERCC3** ]  to DNA damage in SNS occurred much less efficiently in p53 Null or p53 compromised cells.  Ref: 12713809 DNA Repair Amst , 2003
- The preferred substrate for SCP1 is RNAP II phosphorylated by TFIIH  [ **GTF2H1** ]   [ **ERCC3** ] .  Ref: 12721286 J Biol Chem< MedlineTA>J Biol Chem, 2003
- Tumor suppressor p53 dependent recruitment of nucleotide excision repair factors XPC and TFIIH  [ **GTF2H1** ]   [ **ERCC3** ]  to DNA damage.  Ref: 12713809 DNA Repair Amst , 2003
- In this study, the regulatory effect of p53, on the spaciotemporal recruitment of XPC and TFIIH  [ **GTF2H1** ]   [ **ERCC3** ]  to DNA damage sites, was investigated in repair proficient and deficient human cells in situ.  Ref: 12713809 DNA Repair Amst , 2003
- The archaeal transcriptional machinery is polII like but does not require ATP or TFIIH  [ **GTF2H1** ]   [ **ERCC3** ]  for open complex formation.  Ref: 12783891 J Biol Chem< MedlineTA>J Biol Chem, 2003
- Taken together, the results demonstrated that p53 plays a pronounced role in the damage recognition and subsequent assembly of repair machinery during GGR and the recruitment of XPC and TFIIH  [ **GTF2H1** ]   [ **ERCC3** ]  to CPD is p53 dependent.  Ref: 12713809 DNA Repair Amst , 2003
- Furthermore, these mutations conspicuously augmented the binding of hTFIIE beta to the p44 subunit of TFIIH  [ **GTF2H1** ]   [ **ERCC3** ] .  Ref: 12665589 Mol Cell Biol, 2003
- Colocalization experiments of the NER factors showed that in normal human cells, XPC and TFIIH  [ **GTF2H1** ]   [ **ERCC3** ]  are rapidly and efficiently recruited to DNA damage within SNS.  Ref: 12713809 DNA Repair Amst , 2003
- The antibody study indicated that they thus altered the conformation of one side of TFIIH  [ **GTF2H1** ]   [ **ERCC3** ] , consisting of p44, XPD, and Cdk activating kinase subunits, that is essential for the transition stage.  Ref: 12665589 Mol Cell Biol, 2003
